# Improvement in daily functioning after aerobic exercise training in schizophrenia is sustained after exercise cessation

**DOI:** 10.1007/s00406-021-01282-8

**Published:** 2021-06-18

**Authors:** Peter Falkai, Isabel Maurus, Andrea Schmitt, Berend Malchow, Thomas Schneider-Axmann, Lukas Röll, Sergi Papiol, Thomas Wobrock, Alkomiet Hasan, Daniel Keeser

**Affiliations:** 1 Department of Psychiatry and Psychotherapy, University Hospital, Ludwig-Maximilians-University Munich, Nussbaumstrasse 7, 80336 Munich, Germany; 2 Laboratory of Neurosciences (LIM-27), Institute of Psychiatry, University of São Paulo (USP), Rua Dr. Ovídio Pires de Campos 785, São Paulo-SP, 05403-903 Brazil; 3 Department of Psychiatry and Psychotherapy, University Medical Center Göttingen, Von-Siebold-Str. 5, 37075 Göttingen, Germany; 4 Institute of Psychiatric Phenomics and Genomics, University Hospital, Ludwig-Maximilians-University Munich, Nussbaumstrasse 7, 80336 Munich, Germany; 5 Department of Psychiatry and Psychotherapy, County Hospitals Darmstadt-Dieburg, Krankenhausstrasse 7, 64823 Gross-Umstadt, Germany; 6 Department of Psychiatry, Psychotherapy and Psychosomatics, Medical Faculty, University of Augsburg, Bezirkskrankenhaus Augsburg, Augsburg, Germany

**Keywords:** Aerobic exercise, Schizophrenia, Daily functioning, Hippocampus, Neuronal plasticity, Neurogenetics

Cognitive deficits in schizophrenia are crucially important for patients’ functional outcome, but they do not significantly improve with currently available treatment options. On the basis of animal work indicating that physical activity stimulates neurogenesis in adults and enhances cognitive performance, several groups around the world performed aerobic exercise studies in schizophrenia (SZ). These studies demonstrated that physical exercise improves negative symptoms and cognitive dysfunction and indicated that the improvements in cognition may be related to improved brain plasticity in relevant brain regions, such as the hippocampus [1]. The neurobiological mechanisms proposed as drivers of these effects include synaptic plasticity and, more recently, oligodendrocyte differentiation [2]. However, unclear was whether these effects on brain plasticity were maintained after cessation of exercise.

**Fig. 1:**
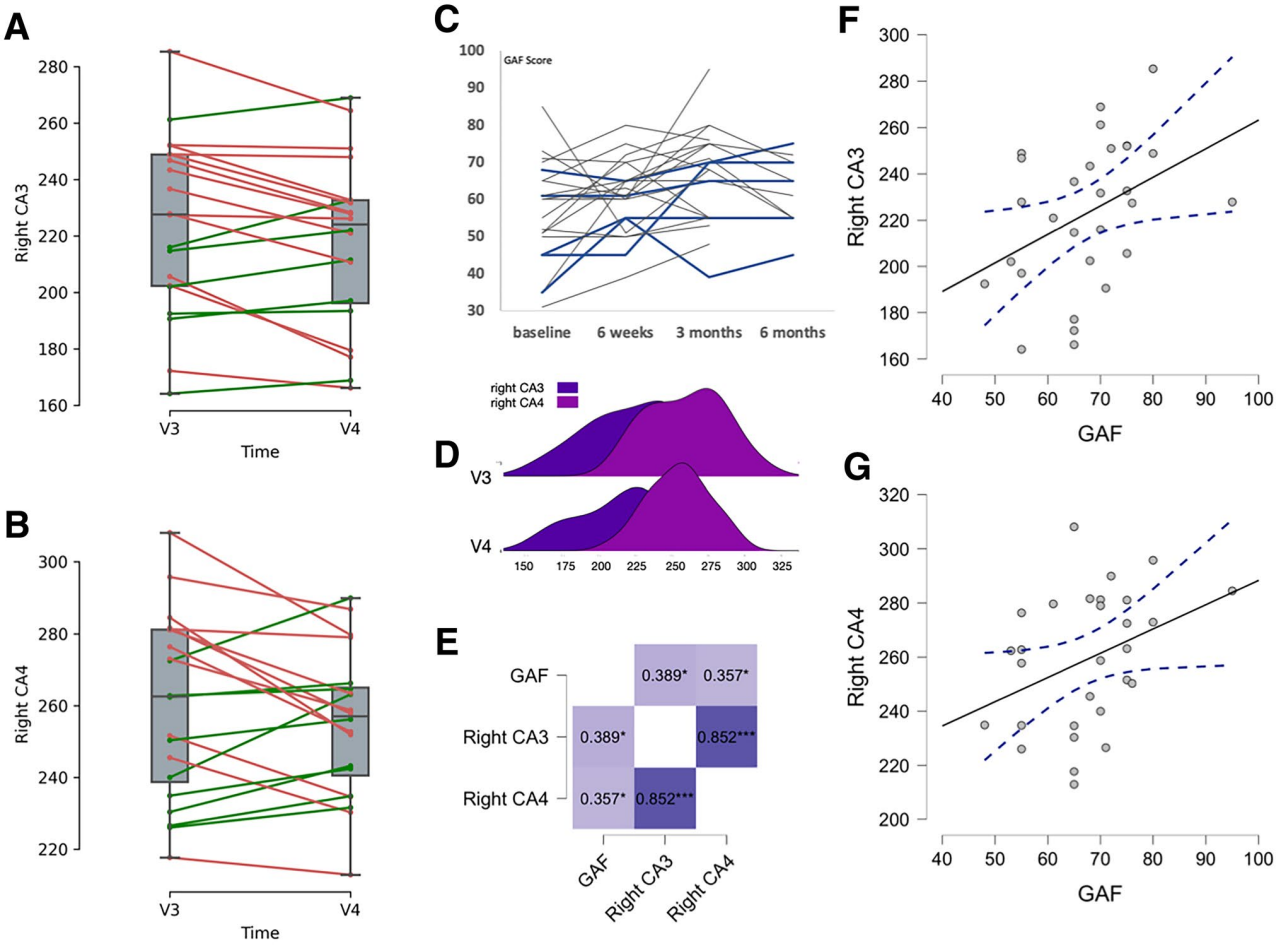
Change from 3 months (V3) to 6 months (V4) in gray matter volume (mL) in the hippocampal subfields of the right cornu ammonis 3 (CA3) (**A**) and CA4 (**B**). Red lines indicate a decrease and green lines indicate an increase. **C** Change in Global Assessment of Functioning (GAF) score in the exercise group from baseline to 6 months. **D** Group distribution of hippocampal subfield volumes for the right CA3 and CA4 at 3 and 6 months. **E** Heatmap plot of correlations between change in volume in right CA3 and CA4 and change in GAF score. **F** Correlation between change in volume of right CA3 and change in GAF score and **G** between change in volume of right CA4 and change in GAF score. The dashed line indicates the 95% CI. *CA* cornu ammonis, *GAF* Global Assessment of Functioning, *V* visit

In a controlled 3-arm study, we compared patients with SZ and controls who performed indoor cycling as aerobic exercise training with patients with SZ who played table soccer; all 3 groups also received cognitive remediation [3, 4]. In patients, the combination of indoor cycling and cognitive remediation significantly improved daily functioning after 3 months as measured by the Global Assessment of Functioning (GAF; Fig. 1C) and the Social Adjustment Scale (SAS II) [3]. When we compared the proportion of patients who showed an improvement of least one level of functioning on the GAF (10 points), we found a significantly higher proportion in the indoor cycling group (45.5%) than in the table soccer group (9.5%; two-sided Fisher’s exact test: *p* = 0.016; odds ratio = 0.126, 95% CI 0.024–0.679). However, an improvement of only one level of functioning on the GAF provides limited information on outcome, because any improvement in GAF scores that are below 65 points after 3 months is not a surrogate marker for recovery [5]. Therefore, in a second step, we compared the proportion of patients who showed an improvement of at least 10 points on the GAF and also had a total GAF score greater than or equal to 65 points after 3 months. In the indoor cycling group, 36.4% of patients fulfilled both criteria, but in the table soccer group, only 9.5% did (nonsignificant in two-sided Fisher’s exact test: *p* = 0.069; odds ratio = 0.184; 95% CI 0.034–1.005).

As outlined in our original paper [3], participants performed the aerobic exercise training for 3 months and were then followed for 3 months to see whether the functional improvement persisted. In the whole group, the improvements seen after 3 months of training were lost after 3 more months [3]. However, when we re-evaluated these results, we found that 45% of the patients maintained or even improved their GAF score beyond month 3 (see Fig. 1C). The improvement was related to a volume increase of the right hippocampal subfields cornu ammonis 3 (CA3) and CA4 (Fig. 1D). Our previous work had already indicated that this volume increase was due to an increase in gray matter in the right temporal pole [4]. At that time, we found no significant group effect in volume change of the hippocampal subfields, but our re-evaluation of the findings showed a clear difference in the patients who responded and in whom effects persisted (Fig. 1A and B). Pearson correlation analyses showed a significant positive correlation between changes on the GAF scale and volume changes in the right CA3 and CA4 (Pearson Correlations: GAF-right_CA3, *r* = 0.39, *p* = 0.02; GAF-right_CA4, *r* = 0.36, *p* = 0.03).

These findings support the notion that neural plasticity can be fostered and maintained by aerobic exercise training in a meaningful proportion of patients with SZ, i.e., in more than 40%. Hypothetically, this subgroup of “exercise responders” could be characterized by their individual schizophrenia genetic risk burden. This notion is particularly interesting in light of the most recent genome-wide association study in SZ, which indicated that the top genetic hits were tightly linked to synaptic disturbances in frontotemporal brain structures, including the hippocampus [6]. Along these lines, our previous studies based on SZ polygenic risk scores (SZ-PRS), a quantitative estimate of genetic risk, showed modulatory effects of SZ-PRS on the effects of exercise on the change in CA4/dentate gyrus volume and the clear role of genes involved in synaptic ion channel activity, calcium signaling, glutamate signaling, and regulation of cell morphogenesis on the association between SZ-PRS and these effects on brain structure [7]. Moreover, our group performed another secondary analysis of cell type-specific PRS and found that the polygenic modulatory effect on CA4/dentate gyrus volume change was especially prominent in analyses of oligodendrocyte precursor cell-specific SZ-PRS or radial glia-specific SZ-PRS, suggesting an important role of these brain cell types in modulating the effects of exercise on hippocampal plasticity [8]. If replicated in a larger sample, these findings would allow us to define a distinct subgroup of patients who respond to and maintain the beneficial effects of aerobic exercise training on cognitive and daily functioning. Presumably, the pathophysiology of this group would rest on a disturbance of brain regenerative mechanisms, in particular regarding synaptic and myelin plasticity [9]. New approaches such as induced pluripotent stem cell-based systems may allow us to gain mechanistic insight into these complex biological processes. Such approaches could pave the way for the discovery of candidate biomarkers that can be used to identify those patients who will respond to exercise and for the testing of new or repurposed drug targets in this subgroup of patients.

These results support the importance of longitudinal observation of subgroups or even individual patients. Some researchers are rightly demanding that analyses should be conducted explicitly at both the individual and the group level to enable development of advanced models of generalizability [10]. Our results need further validation in larger samples, and we plan to test them in a multicenter longitudinal follow-up study.
